# Selection and Validation of Reference Genes for RT-qPCR Analysis in Tibetan Medicinal Plant Saussurea Laniceps Callus under Abiotic Stresses and Hormone Treatments

**DOI:** 10.3390/genes13050904

**Published:** 2022-05-18

**Authors:** Huan Liu, Yaning Lu, Xiaojing Wang, Xiaowei Wang, Rongchen Li, Cunfu Lu, Xiaozhong Lan, Yuzhen Chen

**Affiliations:** 1 National Engineering Research Center of Tree Breeding and Ecological Restoration, Key Laboratory of Genetics and Breeding in Forest Trees and Ornamental Plants, Ministry of Education, The Tree and Ornamental Plant Breeding and Biotechnology Laboratory of National Forestry and Grassland Administration, College of Biological Sciences and Technology, Beijing Forestry University, Beijing 100083, China; lhliveseriously@126.com (H.L.); luyaning0321@163.com (Y.L.); wangxj0508@bjfu.edu.cn (X.W.); 15613278310@163.com (R.L.); lucunfu@bjfu.edu.cn (C.L.); 2 Tianjin Institute of Industrial Biotechnology, Chinese Academy of Sciences, Tianjin 300308, China; wangxiaow@tib.cas.cn; 3 The Provincial and Ministerial Co-Founded Collaborative Innovation Center for R & D in Tibet Characteristic Agricultural and Animal Husbandry Resources, The Center for Xizang Chinese (Tibetan) Medicine Resource, Joint Laboratory for Tibetan Materia Medica Resources Scientific Protection and Utilization Research of Tibetan Medical Research Center of Tibet, Tibet Agriculture and Animal Husbandry University, Nyingchi 860000, China

**Keywords:** *Saussurea laniceps*, reference gene, abiotic stress, hormonal treatment

## Abstract

Real-time quantitative PCR (RT-qPCR) is an important technique for studying gene expression analysis, but accurate and reliable results depend on the use of a stable reference gene. This study proposes to test the expression stability of candidate reference genes in the callus of *Saussurea laniceps*, a unique Tibetan medicinal plant. Based on the *S. laniceps* callus transcriptome, eleven candidate reference genes, including *TUA2*, *TUB3*, *TUB8*, *TIF3B1*, *TIF3H1*, *ELF5A*, *PP2AA2*, *UEV1D*, *UBL5*, *UBC36*, and *SKIP1*), were validated for RT-qPCR normalization in the callus under abiotic stress (salt, cold, and UV) and hormone treatments (abscisic acid, MeJA, and salicylic acid). The stability of the candidate genes was evaluated in all the samples of *S. laniceps.* Comprehensive analysis of all samples showed that the best reference genes were *UBC36* and *UBL5*. *ELF5A* and *TIF3B1* were ranked as the most stable genes in the sample sets under abiotic stress. For hormone stimulation, *UBC36* and *TIF3H1* genes had the best stability. This study provides useful guidelines and a starting point for reference gene selection for expression analysis using RT-qPCR techniques in *S. laniceps*.

## 1. Introduction

*Saussurea laniceps* is endemic to the Himalayas, where the alpine environment is very harsh, including dramatic daily temperature changes, limited rainfall, and high-intensity solar radiation; hence, the wild *S. laniceps* population is limited. Moreover, as an important Tibetan folk traditional medicinal species [1,2], market demand also leads to pressure on the wild populations of *S. laniceps*. Damage to or the collection of individual plants and habitat disturbance or loss threaten the long-term survival of *S. laniceps*. Accordingly, the development of protocols for the ongoing protection of this species is essential. Previously, we established efficient in vitro micropropagation methods for *S. laniceps,* both in the callus and plantlets [3,4]. Furthermore, proteomic analysis provides a unique insight into the freezing resistance mechanism of *S. laniceps* callus after cold acclimation [3]. Recently, we found that the color of the callus changes obviously after cold acclimation (4 °C), from ivory (0 d) to pink (6 d), and finally, to red. Chemical evaluation indicated that cold treatments significantly increased the accumulation of flavonoids in *S. laniceps* callus.

Some structural genes related to flavonoid synthesis and transport have been obtained based on the analysis of the *S. laniceps* transcriptome and offer a valuable opportunity to unravel the molecular mechanism underlying flavonoid accumulation in *S. laniceps* (unpublished data). Consequently, the above results imply that propagation of the callus under control situations, such as abiotic stress or hormone treatments, could be an effective approach for producing medicine ingredients instead of wild *S. laniceps*. However, the absence of genetic and genomic tools for this species limits its current utility. Therefore, it is necessary to investigate the molecular mechanism of *S. laniceps* in response to different environmental stimuli.

Detecting gene expression dynamics is a crucial tool for studying plant molecular mechanisms, and real-time quantitative PCR (RT-qPCR) is one approach for doing so [5,6]. It is very effective to introduce internal reference genes to normalize gene expression data [7]. It follows that the expression level of the ideal reference gene should remain constant in the investigated samples [8,9]. Housekeeping genes are mainly essential components of the organelle skeleton (e.g., *ACT*, *TUB*) or involved in the basic biochemical metabolic processes of the organism (e.g., *GAPDH*, *UBQ*), and they are stably and consistently expressed in the vast majority of cells and tissues [10,11], so housekeeping genes are often used as internal reference genes. Unfortunately, an increasing amount of experimental evidence has shown that several of these genes fluctuate depending on the experimental settings [12,13]. Among the many internal reference genes, the selection of a suitable reference gene based on its expression stability in the experimental system is the key to standardization.

The purpose of this work is to uncover stable and reliable candidate reference genes from *S. laniceps*. Eleven genes (*TUA2*, *TUB3*, *TUB8*, *TIF3B1*, *TIF3H1*, *ELF5A*, *PP2AA2*, *UEV1D*, *UBL5*, *UBC36*, and *SKIP1*) were selected from the *S. laniceps* callus transcriptome, and the expression levels of these genes were determined using RT-qPCR under abiotic stresses (salt, cold, and UV) and hormonal stimulation (abscisic acid (ABA), MeJA, and salicylic acid (SA)). Finally, the most and least stable genes were used to assess the expression pattern of three target genes (*SlPAL*, *SlUGT84A1*, and *SlOPR*) to check the correctness of the proposed reference genes. These results lay the basis for further research on the gene regulation mechanism in *S. laniceps*.

## 2. Materials and Methods

### 2.1. Plant Material

The induction and culturing of *S. laniceps* calluses were performed as described previously [3]. The calluses, grown on MS medium for 15 days and in good growth condition, were treated according to the following process. During hormone treatment, the calluses were transferred to MS agar medium supplemented with 50 uM MeJA, 24 uM SA, and 75.7 uM ABA for 0, 4, 10, 20, and 25 h, respectively. For cold treatments, the calluses were kept at 4 °C in a refrigerator for 0, 3, 6, and 9 d. For UV irradiation, the calluses were exposed to UV-B (313 nm) light for 0, 10, 24, 32, or 55 h. For salt treatment, the calluses were cultured on MS agar medium containing 100 mM NaCl for 0, 2, 3, 4, 5, or 7 d. Samples were collected after different treatments. All samples were collected in three biological replicates. Before RNA extraction, all samples were promptly frozen in liquid nitrogen and kept at −80 °C.

### 2.2. Total RNA Extraction and Complementary DNA Synthesis

Following the manufacturer’s instructions, total RNA was extracted from samples using the Plant RNA Extraction Kit (Takara, Japan) and treated using Transcript One-Step gDNA Removal. RNA integrity was verified on 1% agarose gel. To quantify the amount and purity of total RNA, a spectrophotometer (Thermo Fisher Scientific, Waltham, MA, USA) was used to detect absorbances at 260/280 and 260/230 nm; the following analyses were limited to RNA samples with absorbance at an A260/A280 ratio between 1.8 and 2.0 and an A260/A230 ratio >2.0; only RNAs that met the criteria were used for subsequent cDNA synthesis. cDNA Synthesis SuperMix cDNA (TransGen Biotech, Beijing, China) with oligo (dT) primers was used to reverse-transcribe total RNA (1μg) into cDNA. Then, the cDNA samples were stored at −20 °C until used as RT-qPCR templates.

### 2.3. Designing Primer and Testing Performance

In our previous study, RNA-seq of the *S. laniceps* callus acclimated at 4 °C for 0, 6, and 9 d was performed. Eleven stably expressed genes were screened from the database of RNA-seq of *S. laniceps* callus on the condition of FPKM > 50 and log_2_ fold-change less than 0.01. According to the principle of RT-qPCR primer design, through NCBI Primer-BLAST and Oligo7.0 software, RT-qPCR primers of 11 internal reference genes were designed, including *TUB8* (*β-tubulin 8*), *TUB3* (*β-tubulin 3*), *TUA2* (*α-tubulin 2*), *TIF3B1* (*translation initiation factor 3B*), *TIF3H1* (*translation initiation factor 3H1*), *ELF5A* (*Elongation factor 5A*), *PP2AA2* (*protein phosphatase 2A*), *SKIP1* (*SKP1 interacting protein 1*), *UBC36* (*ubiquitin conjugation enzyme 36*), *UBL5* (*ubiquitin-like protein 5*), and *UEV1D* (*ubiquitin E2 variant 1D*). The length of the primers was 19–22 bp, and the length of the amplification products was between 101 and 217 bp. The primer was synthesized by the TSINGKE company (Beijing, China). The gene features and primer information are listed in Table 1.

cDNA was diluted in a tenfold gradient (1, 1/10, 1/100, and 1/1000) and used to make a standard curve and, thus, obtain the correlation coefficient (*R*^2^) and amplification efficiency (*E*) for all primer pairs, and amplification and melting curve analyses were utilized to determine the specificity of each primer pair.

All RT-qPCR procedures were done in 96-well plates with Quant-Studio^TM^ Real-Time PCR Software (Applied Biosystems, Waltham, MA, USA) and SuperReal PreMix Plus Mix (Tiangen, China). Each 10 μL reaction included: 5.0 μL of 2 × SuperReal PreMix Plus Mix, 0.3 μL of each primer (10 μM), 1.0 μL cDNA, and 3.4 μL of RNase-free H_2_O. The protocol for RT-qPCR was as follows: 95 °C for 15 min, then 40 cycles of 10 s at 95 °C, 60 °C for 30 s, then 72 °C for 20 s. After 40 cycles of testing the specificity of each primer pair across a temperature range of 60–95 °C within 15 min, the melting curve was created. This established that the fluorescence was due to a single PCR product and not primer dimers or a non-specific product generated during PCR. For each sample, three technical replicates were employed.

### 2.4. Data Analysis and Evaluation

The CT values indicate each candidate reference gene’s expression level. The effectiveness of amplification was estimated using the formula Efficiency (%) = (10^−1/slope^ − 1) × 100% [14]. Gene expression stability was calculated by five algorithms: the ΔCT method [15], geNorm [16], NormFinder [17], BestKeeper [18], and RefFinder [19]. The average standard deviation (SD) of all pair-wise pairings of potential reference genes was calculated using the ΔCT method to rank the genes; therefore, the most stable reference gene is the one with the lowest SD [15]. Genes with the lowest M-values, which geNorm calculates for each gene, have the most steady expression. geNorm provides advice that the numbers of reference genes should be applied to standardize the target genes. V_n/n+1_ < 0.15 is a threshold value that shows that even adding more reference genes does not significantly contribute to normalization. V_2/3_ < 0.15, for example, indicates that gene expression data may be normalized with just two reference genes [16]. The BestKeeper program calculates gene stability by comparing the standard deviation (SD) and the coefficient of variation (CV) of Ct values; the smaller the standard deviation and coefficient of variation, the better the stability; on the contrary, the larger the standard deviation and coefficient of variation, the worse the stability. The advantage of this program, unlike GeNorm and NormFinder, is that it does not only analyze the stability of expression of internal reference genes but also compares the expression levels of target genes [18]. NormFinder computes each gene’s stability value (SV) that accounts for both intergroup and intragroup interactions; greater stability is indicated by a lower SV [17]. RefFinder is a novel analysis that combines the four computational methods mentioned above; it performs a comprehensive ranking by calculating the geometric mean of the weights of all genes; a lower geometric mean means more stable gene expression [19].

### 2.5. Validation of Selected Candidate Reference Genes

Target gene expression levels (*SlPAL*, *SlUGT84A1*, and *SlOPR*) were analyzed using RT-qPCR under UV and SA treatments to verify the chosen reference genes. Primer creation and detection for these three genes were carried out using the procedures described above. The 2^−^^ΔΔCT^ approach was used to calculate relative expression levels.

### 2.6. Statistical Analysis

Experimental data were presented as means ± standard deviation (SD) of three independent replicates. Based on IBM SPSS Statistics 21 software, the Duncan method of single-factor ANOVA was used to compare the significant level of difference (*p*  <  0.05). GraphPad Prism 9 software was used for data visualization.

## 3. Results

### 3.1. Evaluation of Primer Specificity and Effectiveness

From the transcriptome of *S. laniceps*, eleven putative reference genes (*TUA2*, *TUB3*, *TUB8*, *TIF3B1*, *TIF3H1*, *ELF5A*, *PP2AA2*, *UEV1D*, *UBL5*, *UBC36*, and *SKIP1*) were identified. The designed primer sequences and RT-qPCR effectiveness (*E*) are shown in Table 1. By electrophoresis on 1% (*m*/*v*) agarose gel, the PCR results amplifying *S. laniceps* cDNA showed a single band of the predicted size for each primer pair (Figure S1), and the melting curves indicated single peaks for each gene, which proves the uniqueness of the primers (Figure S2). The RT-qPCR effectiveness (*E*) was between 90.4% (*ELF5A*) and 109% (*TUB3*). Correlation coefficients (*R*^2^) ranged between 0.991 and 0.999 (Table 1), indicating that each primer pair had a high level of specificity and efficiency.

### 3.2. Expression Distribution of Candidate Reference Genes

**Figure 1:**
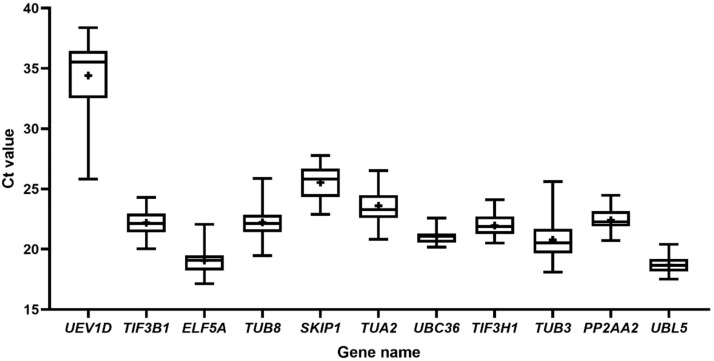
Candidate reference gene expression levels across all samples. The boxes depict the interquartile range. Hyphens above and under the boxes denote the maximum and minimum values, respectively. The median is represented by the line that runs across the box. The mean values are shown by the plus sign in the box. Three technical and biological replicates were carried out in RT-qPCR.

The expression status of the eleven candidate reference genes (*TUB8*, *TUB3*, *TUA2*, *TIF3B1*, *TIF3H1*, *ELF5A*, *PP2AA2*, *SKIP1*, *UBC36*, *UBL5*, and *UEV1D*) was evaluated in 31 samples under abiotic stresses and hormonal treatments using CT values. Boxplots were utilized to show the raw CT values (Figure 1), and Table S2 shows the detailed values. The boxplot of CT values across all samples shows a large range, from 17.53 (*UBL5*) to 38.39 (*UEV1D*). As gene expression and CT value are inversely associated, among the eleven genes, *UBL5* had the lowest mean CT value (18.71), which means its expression levels were the highest, while *UEV1D* was expressed in the lowest abundance gene with the highest mean CT value (34.41). These findings revealed that candidate reference genes did not steady express under all conditions, highlighting the importance of identifying acceptable reference genes in *S. laniceps* under particular experimental circumstances.

### 3.3. Candidate Reference Gene Expression Stability

At first, each reference gene was examined independently in six experimental groups in our investigation. For a more in-depth analysis, the sets were separated into three groups, i.e., (1) Abiotic stress (salt, UV, and cold), (2) Hormone stimuli (ABA, MeJA, and SA), and (3) All (all experimental sets). Then, five analytical methods (ΔCT method, geNorm, NormFinder, BestKeeper, and RefFinder) were used to evaluate the expression stability of the selected internal reference genes.

**Figure 2:**
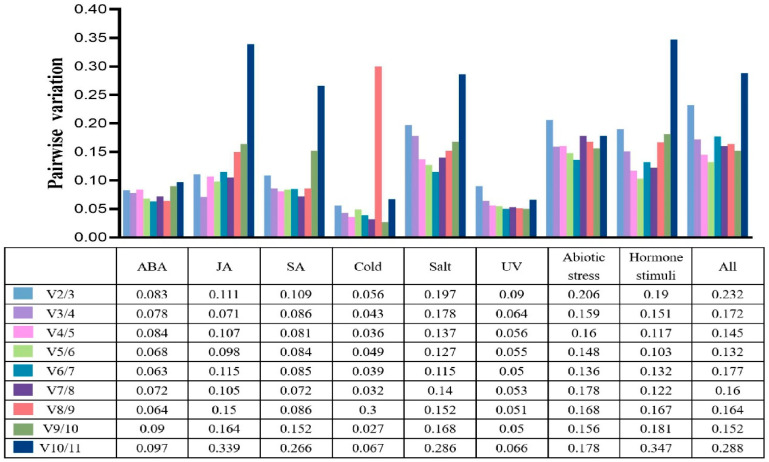
The suitable number of reference genes for normalization in *Saussurea laniceps.* The upper histogram has a corresponding relationship with the data in the lower table; each color bar represents a V_n/n+1_ value.

For RT-qPCR normalization, geNorm supplies a suitable number of reference genes, in which the standard value of V_n/n+1_ is 0.15. V_2/3_ values for the ABA, MeJA, SA, UV, and cold groups were less than 0.15 (Figure 2), demonstrating that two reference genes were sufficient to normalize the expression of the target gene. During salt treatment, hormone stimulation, and all experiments, four reference genes were proposed for normalization based on V_4/5_ values less than 0.15, as determined by geNorm analysis. Meanwhile, in abiotic stress conditions, reliable normalization of gene expression requires five reference genes.

**Figure 3:**
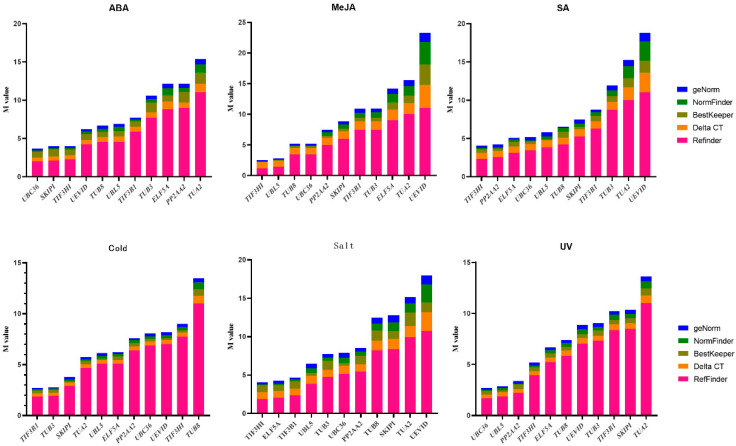
Gene expression stability stacking diagram ranked by five types of analysis methodology under individual experimental conditions. The top three figures are the expression stability ranking of each candidate internal reference gene under hormone treatment, and the bottom is abiotic stress.

As shown in Figure 3, under ABA and UV treatment, *TUA2* was shown to be the most unstable candidate reference gene when evaluated using the four approaches (ΔCT, Normfinder, geNorm, and BestKeeper), while *UBC36* was considered to be the most stable gene. Under salt, SA, and MeJA treatments, *TIF3H1* had the highest expression stability, while *UEV1D* was the least stable. *TIF3B1* was the most optimal internal reference gene in a cold environment, while *TUB8* was the lowest-performing gene.

The ranking order varies in different abiotic stress (Table S1). ELF5A and TIF3B1 had the lowest M-values (0.333) in the abiotic stress category, followed by *UBL5* (0.532), implying that these were the three best-performing genes in terms of stability in the geNorm analysis. In the NormFinder and ΔCT analyses, they were likewise the two top-ranking genes. However, *UBC36* was the best choice for normalization in the BestKeeper analysis. According to RefFinder’s thorough analyses, the reference gene *ELF5A* was found to be the most stable. *TIF3H1* was likewise the top-ranked gene for the salt treatment, followed by *ELF5A*, *TIF3B1*, and *UBL5*, all of which were associated with abiotic stress. Under the cold treatment, *TIF3B1* and *TUB3* were designated as the two most stable reference genes; however, *UBL5* and *ELF5A* did not make the list of the three most stable genes. When it came to the UV treatment, *UBC36* had the best stability, followed by *UBL5* and *PP2AA2* (Figure 3).

For hormone stimuli, *UBC36* and *TIF3H1* were the best reference genes, followed by *TUB8* and *PP2AA2*, in the geNorm analysis. Surprisingly, similar results were obtained in the remaining three test methods, including ΔCT, NormFinder, and BestKeeper. Therefore, in the RefFinder analysis, *UBC36* and *TIF3H1* were the two ideal reference genes when all hormonal stimuli were taken into account (Table S1). *TIF3H1* and *PP2AA2* had the highest consistency when it came to the SA treatment; under the MeJA treatment, *TIF3H1* and *UBL5* were determined to be the most trustworthy reference genes. *UBC36* and *SKIP1* were the most stable reference genes under ABA stimuli, whereas *PP2AA2* and *TUA2* were the most unstable, which differed from other hormone treatments (Figure 3).

In geNorm, BestKeeper, and NormFinder tests, *UBC36* and *UBL5* ranked first and second, respectively, when all of the samples were evaluated. In the ΔCT analysis, however, *UBC36* remained the best internal reference gene, but *UBL5* was replaced by *PP2AA2*. By using RefFinder, the eleven reference genes were graded from best to lowest stability as: *UBC36* > *UBL5* > *PP2AA2* > *TIF3B1* > *TIF3H1* > *ELF5A* > *TUA2* > *SKIP1* > *TUB8* > *TUB3* > *UEV1D*. Finally, among all *S. laniceps* samples, *UBC36* and *UBL5* were the two most ideal internal reference genes (Table S1).

### 3.4. Validation of Candidate Reference Genes

**Figure 4:**
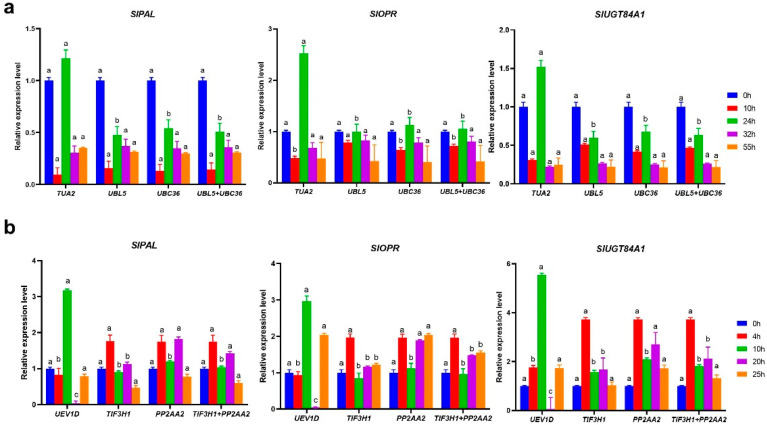
Relative expression patterns of target genes. (**a**) UV treatment, (**b**) SA treatment. The most stable reference genes (*UBL5* and *UBC36* for UV; *TIF3H1* and *PP2AA2* for SA), and unstable genes (*TUA2* for UV and *UEV1D* for SA) were selected for normalizing RT-qPCR data. Data are provided as mean ± SD; all RT-qPCR procedures were performed in triplicate for technical and biological replicates. Note: Different lowercase letters indicate significant differences at 0.05 levels.

To guarantee the correctness and reliability of our findings, the relative expression tendency of *SlPAL*, *SlUGT84A1*, and *SlOPR* was evaluated under hormone stimulus (SA) and abiotic stress (UV). The top two ranked internal reference genes under the SA and UV treatments, respectively (*UBC36* and *UBL5* for SA; *TIF3H1* and *PP2AA2* for UV), were chosen, as well as one unstable gene (*UEV1D* for SA and *TUA2* for UV). As shown in Figure 4, when *TIF3H1* and *PP2AA2* were employed for normalization, the expression levels of *SlPAL*, *SlUGT84A1*, and *SlOPR* exhibited similar changing patterns. Contrary to that, when *UEV1D* was utilized as a normalizer under the SA treatment, the expression trends of *SlPAL*, *SlUGT84A1*, and *SlOPR* were altered, and the expression profile of target genes at the 10 and 20 h stages were abnormally calculated. Simultaneously, under the UV treatment, when using stable reference genes for normalization, the expression tendency of *SlPAL*, *SlUGT84A1*, and *SlOPR* were essentially the same, while when *TUA2* was used, the relative expression levels at 20 h were overestimated.

## 4. Discussion

As a powerful analytical technique, RT-qPCR has emerged as the standard method for gene expression studies [20]. However, reliable and accurate gene transcript quantification depends on the use of stable reference genes [21]. Under ideal conditions, the expression levels of the internal reference genes are considered to be more stable across tissues or various environments in the organism [22]. However, an increasing number of studies demonstrate that reference genes vary significantly because they are involved in a variety of cellular functions in addition to sustaining fundamental cellular processes [23].

In the present work, eleven alternative internal reference genes were selected from the transcriptome of *S. laniceps*, and their expression stability under different stress environments was validated. Because the four computational approaches used to rate the stability of the candidate genes are based on diverse ideas and approaches, the findings produced were not entirely consistent. During our investigation, we discovered that the rankings produced by ΔCT, geNorm, and NormFinder were comparable but distinct from those produced by BestKeeper [24], which were consistent with Mallona’s and Wang’s findings [25,26]. For example, under salt treatment, the results of rankings calculated by ΔCT, geNorm, and NormFinder were almost identical, with only slight differences, which were different from the results estimated by BestKeeper. As a consequence, by merging the findings of the four computational approaches, RefFinder was utilized to provide a thorough ranking list for the candidate reference genes [19,27].

Our results showed some differences in the stability of the endogenous genes under different treatments, and none of the reference genes were stable under all treatment conditions, so it is required to test the stability of the internal reference genes before performing RT-qPCR. *TIF3H1* and *UBC36* expression stability were optimal under multiple treatment conditions, especially under hormone treatments. For example, under the MeJA and SA treatments, the stability of *TIF3H1* was ranked first. Meanwhile, the stability ranking of *TIF3H1* was in the top four except under cold treatment. Nevertheless, *TIF3B1* showed great variability under a variety of treatment conditions. In a reference screen, Zhao et al. concluded that *TIF1α* is stable at multiple growth stages in a variety of tissues of *Anemone flaccida Fr. Schmidt* [28], and Saha et al. confirmed that *TIF* is stable in *Pisum sativum* under cold stress [29]. The reason for such different results may be due to the different expressions of different *TIF* genes in different species. *UBC* was demonstrated to have low stability in rice cultivated under various environmental circumstances, while it was the best in *Platycladus orientalis* throughout all phases of growth and in all stressful situations [30]. Our result showed that although *UBC36* did not perform well under cold stress and salt stress, its expression was relatively stable in overall performance.

In the present study, some genes that are not much used as internal reference genes showed better stability under certain conditions. Løvdal et al. found that tomato *PP2AA2* had better stability under the cold treatment [31]. However, in our study, *PP2AA2* showed lower stability under the ABA treatment but good stability under the SA treatment. In *Stipa grandis*, *SKIP5* had the lowest M-value under cold treatment, and the most unstable gene was *SKIP11* under multiple treatments [32]. Yang et al. revealed that in some experimental settings, *SKIP5-1* and *SKIP5-2* showed good stability in *Caragana korshinskii* [33], while we found that *SKIP1* showed good stability under ABA and low-temperature treatments but not under the other four treatments. *UBL5* is an atypical ubiquitin-like protein, and its stability is moderate under various treatment conditions [34]. Similarly, our experimental results showed that *UBL5* had relatively good stability under some conditions, such as the MeJA treatment and UV stress.

On the other hand, some genes that are considered to be traditional internal reference genes did not perform as stably as expected in the current study. Actin is a highly conserved multifunctional protein that exists in almost all eukaryotic cells, so it is commonly used as a reference gene to normalize target genes [35,36]. However, in our previous pre-experiments, we found that the expression of the actin gene was low under some conditions, and it did not show a trend of stable expression, which is similar to the study in *Haloxylon ammodendron* [37]. Hence, actin was not included in this study. *TUA2*, *TUB3,* and *TUB8,* belonging to the tubulin family, have been extensively used as internal reference genes in RT-qPCR analysis because they are highly stable in many plants, including *Hedysarum coronarium* [38]. However, our study showed that *TUA2* and *TUB8* were not as stable as expected. For example, under ABA and UV treatments, *TUA2* was the most unstable expression gene, and *TUB8* showed the most unstable expression trend under the low-temperature treatment. Similarly, the stability of *TUA6* in cold shock treatment was poor in *Panicum virgatum* [22], and *TUA* and *TUB* had significant expression variation under different temperature conditions and ABA treatment in *Caragana korshinskii* [33]. In the present study, *ELF5A* showed different stability values under different stimulation conditions; for example, its expression stability was better under the salt treatment and the SA treatment and was poorer under ABA and MeJA treatments. Similarly, Li et al. [39] found that the expression stability of *ELF1-α* was poor in *Glehnia* *littoralis* under ABA and MeJA treatments, whereas, in virus-infected *Arabidopsis thaliana*, *ELF1-α* had good stability [40]. Finally, our study showed that *UEV1D*, which belongs to the ubiquitin family and is mainly involved in the process of DNA damage repair [41], showed the trend of unstable expression under most stimulation conditions.

In conclusion, to the best of our knowledge, this is the first study aimed at validating a set of candidate reference genes for gene expression normalization using RT-qPCR in *S. laniceps*. In the present research, 11 candidate reference genes were identified in the transcriptome database of *S. laniceps,* and their expression stability was evaluated across a set of all samples using the computer programs ΔCT, geNorm, NormFinder, and BestKeeper. The internal reference gene’s stability varied depending on the treatment. *ELF5A* and *TIF3B1* showed good stability under abiotic stress, and their reliability and effectiveness have been verified by *SlPAL*, *SlUGT84A1*, and *SlOPR*. Moreover, for hormone stimulation, *UBC36* and *TIF3H1* genes had the best stability. *UBC36* and *UBL5* were the most ideal reference genes when all samples were taken into account. These results may constitute a starting point for selecting reference genes in the future for more accurate normalization in different tissues and under other experimental conditions in *S. laniceps*.

## Figures and Tables

**Table 1:** Primer sequences of eleven potential reference genes; RT-qPCR effectiveness (*E*) in *Saussurea laniceps*.

Gene	Gene Description	Primer Sequence (5′-3′)	Product (bp)	*E* (%)	*R* ^2^		Arabidopsis Ortholog
		Forward/Reverse					
*TUA2*	Tubulinalp α-2 chain	F-TCAGGCCGGGGTATTCAGGTTG	146	94.5	0.998		AT1G50010
		R-CAGCACCAGTTTCGCTGAAG					
*TUB3*	Tubulinalp β chain 3	F-AGATCTGGTGCCTACGGACA	104	109	0.999		AT5G62700
		R-TCAGCGCCTTCGGTATAGTG					
*TUB8*	Tubulinalp β 8	F-TGGTACACTGGTGAAGGAATGG	107	94.6	0.995		AT5G23860
		R-TCATCAGCAGTCGCATCTTGA					
*TIF3B1*	Translation initiation factor 3B	F-AATCCGAGGCCCGATGTTAG	141	108	0.998		AT5G27640
		R-GAATCCCTTCAACCCAGCCA					
*TIF3H1*	Translation initiation factor 3 subunit H1	F-TCGGGGAGAGGCTTGAAGAT	141	101	0.998		AT1G10840
		R-CCGTAATCTGTCACGTCAGC					
*ELF5A*	Elongation factor 5A	F-ATGTCGGACGAAGAGCATCA	101	90.4	0.993		AT1G69410
		R-ACGATGTGACCTCCTTTGCG					
*PP2AA2*	Protein phosphatase 2A	F-TGCCTATCCTCCGCAAGTTC	123	97.4	0.998		AT3G25800
		R-TGAGTTTCCAGATGTTCGCCT					
*UEV1D*	Ubiquitin E2 variant 1D	F-GGCACTATCATTGGCCCTCA	101	93.6	0.992		AT3G52560
		R-CGCACACTAGGAGGCCCTCTC					
*UBL5*	Ubiquitin-like protein 5	F-GACGATACCATCGGCGACTT	141	90.5	0.998		AT5G42300
		R-GCCCATGCCATCGTGAATCT					
*UBC36*	Ubiquitin-conjugating enzyme 36	F-GACGAGTCCACTCCTTTGCT	151	92.3	0.991		AT1G16890
		R-AGCCCTGCTCTTCAGATTCG					
*SKIP1*	SKP1 interaction partner 1	F-GCAGACAAACATGGCACCTC	106	96.2	0.997		AT5G57900
		R-CTCAGGACTCTACTCCGGCT					

## Data Availability

Not applicable.

## Supplementary Materials

The following supporting information can be downloaded at: https://www.mdpi.com/article/10.3390/genes13050904/s1, Figure S1: Electrophoresis detection of RT-qPCR amplification products of 11 internal reference genes of *Saussurea Laniceps:* (1) *SKIP1*; (2) *UBL5*; (3) *TUB3*; (4) *PP2AA2*; (5) *ELF5A*; (6) *TIF3H1*; (7) *UEV1D*; (8) *UBC36*; (9) *TUA2*; (10) *TUB8*; (11) *TIF3B1*; Figure S2: Melting curves for eleven reference genes in Saussurea Laniceps; Table S1: Gene expression stability under multiple conditions ranked by ΔCT, BestKeeper, NormFinder, geNorm, and RefFinder; Table S2: Raw Ct values of 11 candidate reference genes under different treatments with 3 replicates.
