# Supplementary material for: Selection and Validation of Reference Genes for RT-qPCR Analysis in Tibetan Medicinal Plant Saussurea Laniceps Callus under Abiotic Stresses and Hormone Treatments
Source: Genes (Basel). 2022 May 18;13(5):904. doi: 10.3390/genes13050904 (PMC9140610; doi:10.3390/genes13050904)
Supplement: Supplementary file 1 [file genes-13-00904-s001.zip › genes-1707782-supp/genes-1707782-supp1.pdf]

Selection and validation of reference genes for RT-qPCR analysis in Tibetan medicinal plant *Saussurea laniceps* callus under abiotic stresses and hormone treatments

Huan Liu[1†], Yaning Lu[1†], Xiaojing Wang[1], Xiaowei Wang[3], Rongchen Li1, Cunfu Lu[1], Xiaozhong Lan[2*], and Yuzhen Chen[1*]

1 College of Biological Sciences and Technology, National Engineering Laboratory for Tree Breeding, Beijing Forestry University, Beijing, 100083, China

2 The Provincial and Ministerial Co-founded Collaborative Innovation Center for R&D in Tibet Characteristic Agricultural and Animal Husbandry Resources, The Center for Xizang Chinese (Tibetan) Medicine Resource, Joint Laboratory for Tibetan Materia Medica Resources Scientific Protection and Utilization Research of the Tibetan Medical Research Center of Tibet, Tibet Agriculture and Animal Husbandry University, Nyingchi of Ti-bet, 860000, China

3 Tianjin Institute of Industrial Biotechnology, Chinese Academy of Sciences, Tianjin, 300308, China

* Correspondence: Author to whom correspondence should be addressed.

† These authors contributed equally to this work

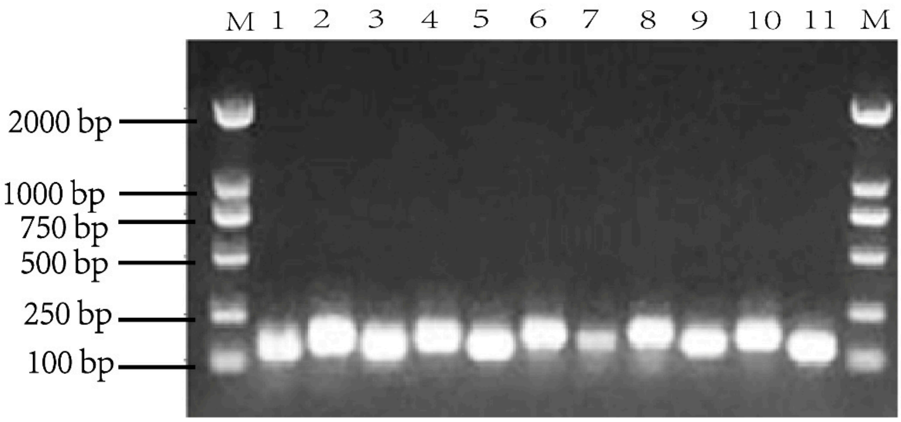

Figure S1. Electrophoresis Detection of RT-qPCR Amplification products of 11 Internal reference genes of *Saussurea Laniceps*

(1)*SKIP1*; (2)*UBL5*; (3)*TUB3*; (4)*PP2AA2*; (5)*ELF5A*; (6)*TIF3H1*; (7)*UEV1D*; (8)*UBC36*; (9)*TUA2*; (10)*TUB8*; (11)*TIF3B1*

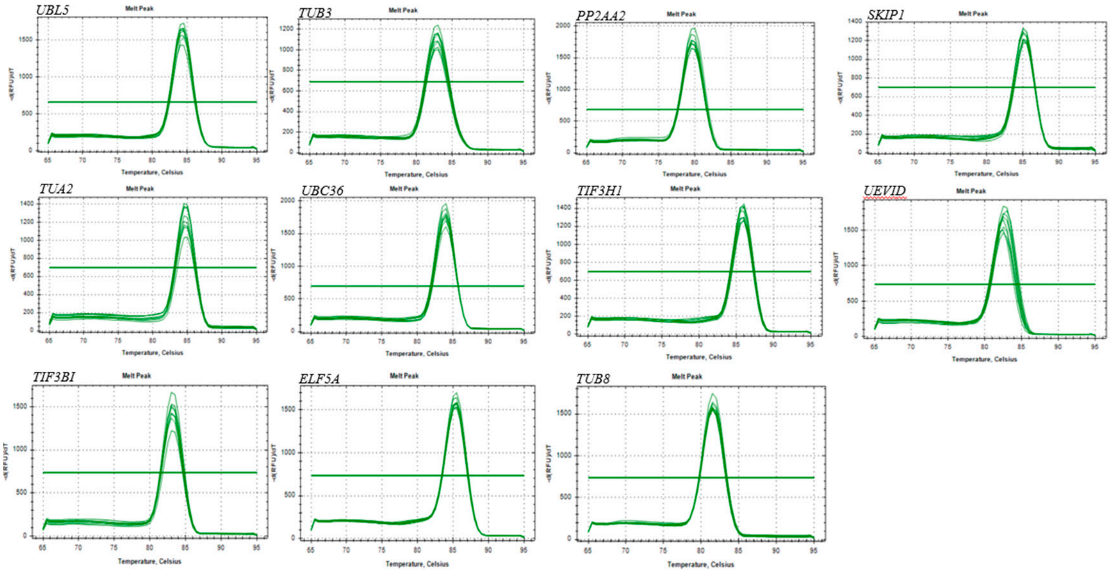

Figure S2. Melting curves for eleven reference genes in *Saussurea Laniceps*

Table S1. Gene expression stability under multiple conditions ranked by △CT, BestKeeper, NormFinder, geNorm and RefFinder.

Groups	Ranking	RefFinder		△CT		BestKeeper		Normfinder		geNorm	
		**Gene**	**Stability**	**Gene**	**Stability**	**Gene**	**Stability**	**Gene**	**Stability**	**Genes**	**Stability**
**Abiotic stress**	1	*ELF5A*	1.41	*ELF5A*	1.05	*UBC36*	0.34	*ELF5A*	0.10	*TIF3B1*	0.33
	2	*TIF3B1*	2.11	*TIF3B1*	1.11	*UBL5*	0.57	*TIF3B1*	0.35	*ELF5A*	0.33
	3	*UBL5*	2.71	*UBL5*	1.12	*TIF3HI*	0.65	*UBL5*	0.42	*UBL5*	0.53
	4	*UBC36*	2.83	*UBC36*	1.16	*ELF5A*	0.70	*UBC36*	0.51	*UBC36*	0.61
	5	*TIF3HI*	4.40	*TIF3HI*	1.21	*TIF3B1*	0.85	*TIF3HI*	0.65	*TIF3HI*	0.72
	6	*TUA2*	6.45	*TUA2*	1.29	*PP2AA2*	0.94	*TUA2*	0.75	*TUA2*	0.82
	7	*PP2AA2*	6.74	*PP2AA2*	1.39	*UEVID*	0.96	*PP2AA2*	0.99	*PP2AA2*	0.89
	8	*TUB3*	8.24	*TUB3*	1.61	*TUA2*	1.04	*TUB3*	1.36	*TUB3*	1.04
	9	*UEVID*	8.68	*UEVID*	1.79	*TUB3*	1.49	*UEVID*	1.49	*SKIPI*	1.16
	10	*SKIPI*	9.97	*SKIPI*	1.83	*TUB8*	1.59	*SKIPI*	1.66	*UEVID*	1.28
	11	*TUB8*	10.74	*TUB8*	2.05	*SKIPI*	1.61	*TUB8*	1.91	*TUB8*	1.42
**Hormone stimuli**	1	*UBC36*	1.00	*UBC36*	1.17	*UBC36*	0.53	*UBC36*	0.25	*UBC36*	0.50
	2	*TIF3HI*	1.68	*TIF3HI*	1.2	*TIF3HI*	0.56	*TIF3HI*	0.25	*TIF3HI*	0.50
	3	*PP2AA2*	3.94	*PP2AA2*	1.26	*UBL5*	0.57	*UBL5*	0.36	*TUB8*	0.58
	4	*TUB8*	3.94	*TUB8*	1.27	*TUB8*	0.61	*PP2AA2*	0.39	*PP2AA2*	0.63
	5	*UBL5*	4.24	*TIF3B1*	1.3	*PP2AA2*	0.71	*TUB8*	0.40	*TIF3B1*	0.66
	6	*TIF3B1*	5.48	*UBL5*	1.32	*TIF3B1*	0.78	*TIF3B1*	0.47	*UBL5*	0.69
	7	*SKIPI*	7.00	*SKIPI*	1.49	*SKIPI*	0.87	*SKIPI*	0.84	*SKIPI*	0.78
	8	*ELF5A*	8.00	*ELF5A*	1.55	*ELF5A*	0.9	*ELF5A*	0.93	*ELF5A*	0.86
	9	*TUA2*	9.00	*TUA2*	1.92	*TUA2*	1.4	*TUA2*	1.54	*TUA2*	1.02
	10	*TUB3*	10.00	*TUB3*	2.25	*TUB3*	1.51	*TUB3*	2.03	*TUB3*	1.20
	11	*UEVID*	11.00	*UEVID*	3.89	*UEVID*	3.38	*UEVID*	3.81	*UEVID*	1.69
**All**	1	*UBC36*	1.00	*UBC36*	1.23	*UBC36*	0.43	*UBC36*	0.23	*UBC36*	0.50
	2	*UBL5*	2.00	*PP2AA2*	1.24	*UBL5*	0.57	*UBL5*	0.38	*UBL5*	0.50
	3	*PP2AA2*	2.91	*TIF3B1*	1.28	*TIF3HI*	0.69	*PP2AA2*	0.39	*PP2AA2*	0.66
	4	*TIF3B1*	4.12	*UBL5*	1.29	*PP2AA2*	0.78	*TIF3B1*	0.45	*TIF3B1*	0.72
	5	*TIF3HI*	4.40	*TIF3HI*	1.33	*ELF5A*	0.81	*TIF3HI*	0.51	*TIF3HI*	0.77
	6	*ELF5A*	5.73	*ELF5A*	1.41	*TIF3B1*	0.82	*ELF5A*	0.75	*ELF5A*	0.82
	7	*TUA2*	7.24	*TUA2*	1.66	*TUB8*	1.09	*TUA2*	1.19	*TUA2*	0.97
	8	*SKIPI*	8.24	*SKIPI*	1.76	*TUA2*	1.22	*SKIPI*	1.33	*SKIPI*	1.08
	9	*TUB8*	8.45	*TUB8*	1.83	*SKIPI*	1.25	*TUB8*	1.41	*TUB8*	1.20
	10	*TUB3*	10.00	*TUB3*	1.98	*TUB3*	1.31	*TUB3*	1.71	*TUB3*	1.31
	11	*UEVID*	11.00	*UEVID*	3.25	*UEVID*	2.44	*UEVID*	3.12	*UEVID*	1.66
